# Surgical Management of Malignant Pleural Mesothelioma: Impact of Surgery on Survival and Quality of Life—Relation to Chemotherapy, Radiotherapy, and Alternative Therapies

**DOI:** 10.1155/2014/817203

**Published:** 2014-02-03

**Authors:** Sotiris Papaspyros, Sayonara Papaspyros

**Affiliations:** ^1^Cardiothoracic Directorate, Royal Infirmary of Edinburgh, 51 Little France Crescent, Edinburgh EH16 4SA, UK; ^2^Biomedical Sciences, Leeds Metropolitan University, Portland Way, Leeds LS1 3HE, UK

## Abstract

*Introduction.* Malignant pleural mesothelioma (MPM) is an aggressive cancer arising from pleural mesothelium. Surgery aims to either cure the disease or control the symptoms. Two surgical procedures exist: extrapleural pneumonectomy (EPP) and pleurectomy/decortication (P/D). In this systematic review we assess current evidence on safety and efficacy of surgery. *Methods.* Five electronic databases were reviewed from January 1990 to January 2013. Studies were selected according to a predefined protocol. Primary endpoint was overall survival. Secondary endpoints included quality of life, disease-free survival, disease recurrence, morbidity, and length of hospital stay. *Results.* Sixteen studies were included. Median survival ranged from 8.1 to 32 months for P/D and from 6.9 to 46.9 months for EPP. Perioperative mortality was 0%–9.8% and 3.2%–12.5%, respectively. Perioperative morbidity was 5.9%–55% for P/D and 10%–82.6% for EPP. Average length of stay was 7 days for P/D and 9 days for EPP. *Conclusion.* Current evidence cannot definitively answer which procedure (EPP or P/D) is more beneficial in terms of survival and operative risks. This systematic review suggests that surgery in the context of trimodality therapy offers acceptable perioperative outcomes and long-term survival. Centres specialising in MPM management have better results.

## 1. Introduction

### 1.1. Definition and Historical Facts

Malignant mesothelioma is a term used to define aggressive tumours that involve mesothelial cells. These cells normally line body cavities, specifically pleura, peritoneum, pericardium, and testis. They can be localized or diffuse [1]. Worldwide incidence of mesothelioma is increasing. This is probably due to exposure of people to asbestos either knowingly or unknowingly. Asbestos has been proven to be associated with mesothelioma [2].

Historically, malignant mesothelioma was diagnosed in three groups of asbestos-exposed persons [3]. Initially it occurred in miners and millworkers who were directly exposed to asbestos in their work environment. Subsequently, mesothelioma was diagnosed in plumbers, carpenters, defence personnel, and insulation installers. Furthermore people living in industrial areas were inadvertently exposed to asbestos fibres released in the atmosphere [3].

### 1.2. Social and Economic Impact

Malignant mesothelioma in the UK has significant socioeconomic impact. More people die from mesothelioma than from melanoma or cervical cancer [4]. Deaths are expected to peak between 2010 and 2015 [5]. Similar rates are anticipated in Europe [6]. Elsewhere, for example, in countries where asbestos is still unregulated, the prospects are even worse.

There is widespread interest in this disease by doctors and the general public, because millions of people have been exposed to asbestos fibres. Many articles about the dangers of asbestos have been published in newspapers.

In addition to its substantial personal and health care costs, malignant mesothelioma is associated with considerable compensation costs. The predicted total economic burden of malignant mesothelioma related to compensation for asbestos exposure in the next 40 years is up to $200 billion for the United States [7] and $80 billion for Europe [8].

### 1.3. Current Status of Evidence on Management

Malignant pleural mesothelioma (MPM) has caused and is still causing violent debate between protagonists and antagonists of the three options in relation to treatment. Surgery, radiotherapy, and chemotherapy are endlessly discussed, often with limited scientific evidence to support strongly held viewpoints. There is little evidence on how to best manage mesothelioma [9]. A combined effort is needed between respiratory physicians, surgeons, and oncologists. Diagnosis and palliation will be part of the surgeon's role but everyone's big hope and question is “is there a chance of surgical cure?”

### 1.4. Chemo- and Radiotherapy

Surgery has come into play because radiation and chemotherapy have had poor outcomes [10]. The mainstay of chemotherapy is combination treatment with pemetrexed and cisplatin. Other combination therapies have also been used. A number of multicentre studies are still under way [1].

Until recently it was thought that local radiotherapy directed to surgical sites prevents spreading of tumour and radiotherapy can provide relief of chest-wall pain [11]. However, an up-to-date systematic review paper by Price [12] concluded that there is currently no evidence to support the routine role of radiotherapy in patients with mesothelioma.

### 1.5. Surgical Treatment

Surgery became part of management options in the 1970s [13] and large series have been published in relevant journals [14, 15]. The two surgical procedures commonly used are pleurectomy with decortication (P/D) and extrapleural pneumonectomy (EPP). P/D is a more limited procedure and requires less cardiorespiratory reserve; it has a morbidity of 25% and a mortality of 2%. EPP is a more extensive procedure and has a higher mortality around 3.8% [1].

Data from the UK Thoracic Surgical Register of the Society for Cardiothoracic Surgery in Great Britain and Ireland showed that a limited number of patients underwent surgery for mesothelioma in the past decade [5]. Single centre retrospective studies reported favourable results with EPP [15]. In some hospitals, this procedure, within a multimodal treatment strategy, became the standard of care [15].

The largest prospective study to date is the MARS trial. The median survival after EPP within MARS is consistent with 10, 12, 13, and 14 months in larger observational studies. Complication rates were also similar [16].

A best evidence topic paper addressing the question of whether P/D is superior to palliative care in the treatment of patients with malignant pleural mesothelioma came to the following conclusion: “P/D is a morbid operation that is associated with significant peri-operative mortality and complication rates” [17].

### 1.6. Patients' Perspective on the Debate for or against Surgery

The supporters of surgery see their position as giving patients hope. They see the antagonists as pessimistic and lacking in motive.

The antagonists of surgery argue that, when a patient commences trimodality (chemotherapy, radiotherapy, and surgery) treatment, it will take six to nine months to complete it. This is a time during which the patient is in and out of hospital and sometimes travelling long distances from home. They point out an important statement in the Hippocratic oath: “Treatments should not give false hope and needless suffering.”

### 1.7. Objectives of the Current Review

The primary objective of the current review is to determine if surgery improves all-cause mortality and/or quality of life in patients with MPM. We will discuss the different surgical options (EPP versus P/D) and their relevant advantages and disadvantages including mortality and morbidity.

Centres that specialise in the treatment of MPM currently use trimodality therapy. This is a combination of chemotherapy either before (neoadjuvant) or after (adjuvant) surgery, an operation that aims to remove the tumour and radiotherapy.

The evidence around the use of different chemotherapeutic regimes will be discussed as well as the evidence on radiotherapy approaches. The overall success rates of the trimodality therapy will be discussed in relation to survival, freedom from recurrence, and quality of life (symptom relief from breathlessness and pain).

## 2. Methods

### 2.1. Literature Search Strategy

Electronic search was performed of PubMed, Ovid, Cochrane Central Register of Controlled Trials, Cochrane Database of Systematic Reviews, and Web of Science from January 1990 to January 2013. In order to identify all potentially relevant studies we combined “mesothelioma” and “pneumonectomy” or “mesothelioma” and “pleurectomy/decortication” as keywords. We reviewed abstracts from the recent European Association of Cardiothoracic Surgery meetings and I searched the British Thoracic Society website for relevant material.

The reference lists of all retrieved studies were reviewed for further identification of relevant papers. The articles that were found were screened using the inclusion and exclusion criteria (previously described in detail in the protocol of this review).

**Figure 1:**
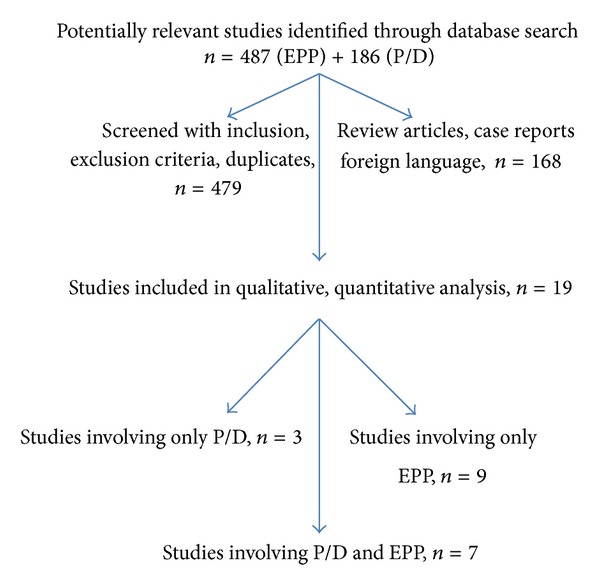
Flowchart of search strategy.

A flowchart of the search strategy is shown in Figure 1.

### 2.2. Selection Criteria

Eligible studies for the present review included those in which patients with histologically proven MPM were treated with surgery (EPP or P/D) either as the only treatment or as part of a protocol involving adjuvant or neoadjuvant chemotherapy and/or radiotherapy.

We did not exclude any forms of chemotherapy or radiotherapy and there was considerable variability between studies in this respect. Patient selection may have varied between institutions and within an institution at different time periods.

All publications were limited to human subjects and English language.

We attempted to identify all relevant randomised controlled trials (RCTs); however, it became clear from the initial search that we would have to include retrospective series and nonrandomised phase I and II trials as the number of RCTs was small. We included studies with more than 20 participants, age greater than 18 (adults), life expectancy at least 12 weeks, and at least moderate performance status either by WHO or Karnofsky criteria.

Abstracts, case reports, conference presentations, and expert opinions were excluded. Review articles were also excluded to avoid duplication of results; however, their references were reviewed.

Two relevant systematic reviews were found. The first was published by Cao et al. [18] and they reviewed the role of EPP in the management of MPP. We reviewed their references and included some of the studies that were relevant to my systematic review. The second was published by Cao et al. [19] and they reviewed the role of trimodality therapy in the management of MPP. We reviewed their references and we also used similar headlines (to theirs) for the summary tables that we constructed. We did not find any systematic review looking at the role of both EPP and P/D in the management of MPP.

Only studies with survival outcomes were included. Few quality-of-life focused studies were identified, as expected. Recurrence-free survival, symptomatic status, and complications were not always reported or measured and we will discuss this below.

## 3. Results

One randomised controlled trial was identified only [16]. The majority of studies were single centre retrospective reviews. Four prospective multicentre trials were included. Two phase II trials were found. There are a number of factors that make interpretation of results difficult. These are discussed below.

The time period that the studies cover extends from 1980 to 2012. Over those two decades the diagnostic modalities have evolved (earlier studies did not have modern scanners available, e.g.), the management has changed from single treatment approach to triple therapy (surgery, chemotherapy, and radiotherapy), the chemotherapeutic agents and radiotherapy strategies have improved or changed, even surgical technique has become more consistent, and surgeons have acquired better skill with experience. Furthermore the postoperative hospital care and support has improved. This evolution has made it possible, in our days, to have diagnosis of mesothelioma at earlier stage and protocol and multidisciplinary team guided management, with better drugs and more skilful surgeons on the background of modern health care institutions with specialist interest. All these factors influence operative mortality and survival, which are the hard endpoints of this review.

The staging system for mesothelioma has changed throughout the years and the definitions of P/D and EPP have only recently started to become more consistent. These factors make meaningful comparison of reported results an extremely difficult and sometimes impossible task.

As the main focus of this review, from an intervention point of view, is surgery for mesothelioma we have classified relevant studies into P/D and EPP. Seven studies included both procedures. We will critically summarise first the studies that include P/D and EPP results, then studies involving only P/D, and lastly studies which focus only on EPP. The characteristics of each publication included as well as their main findings are depicted in the relevant tables. (Tables 1 and 2 for P/D publications and Tables 3 and 4 for EPP publications).

### 3.1. Pleurectomy/Decortication and Extrapleural Pneumonectomy Studies

The study by Schipper et al. [20] was a retrospective review of a single institution over an eighteen-year period. They classified P/D into total and subtotal depending on how extensive the surgical resection was. Patients underwent surgery only. No chemotherapy or radiotherapy was given. They found significantly more complications associated with EPP and higher operative mortality. Moreover patients undergoing EPP were subjected to additional 135 procedures to manage complications. They concluded that overall survival was poor (3-year survival was only 14%). EPP was associated with acceptable mortality and had a median survival significantly better than that for subtotal P/D. They documented their concern regarding the fact that improved survival for EPP came at a cost of high morbidity.

In the paper by Luckraz et al. [21] the surgeon who first described surgical management of pleural mesothelioma in the 1970s (Butchart) is one of the authors. They describe their 30-year experience at their hospital. They did statistical analysis of their results and found that the use of adjuvant therapy (radiotherapy, chemotherapy, or both) was associated with an increased postoperative survival.

On univariate analysis, P/D combined with chemotherapy and radiotherapy was the strongest predictor of prolonged survival. On multivariate analysis EPP alone was an independent risk factor for decreased survival.

An important limitation is that, during the study period, the chemotherapy and radiotherapy regimens changed significantly so it was impossible to make meaningful comparisons. They conclude that despite the serious limitations in their study their overall results represent “real life” situations when comparing EPP to P/D.

Their recommendation was to undertake a randomised controlled trial to determine the relative role of each procedure. (This was done by Treasure et al. in 2006–2010.)

Flores et al. [22] attempted to perform a straightforward comparison of EPP and P/D in their institution, by retrospectively reviewing their results. A large number of patients were reviewed (663) compared to other studies.

The selection of which patient will undergo EPP and which will undergo P/D depended on stage of the MPM, the overall medical fitness of the patient, and requirements of several prospective clinical trials performed during that time period. In patients not participating in protocols that predefined either EPP or P/D, the decision to perform an EPP or P/D was based on surgeons' intraoperative judgement on which procedure was appropriate in order to completely remove the tumour. Also tumour stage and patients' medical condition influenced the decision for EPP or P/D. The patients had chemotherapy or radiotherapy according to which clinical trial they were participating in. The overall survival at 5 years for all patients was poor at 12%. Univariate analysis showed that more aggressive tumour histology and higher stage were associated with significantly worse survival.

A limitation of this study is that the investigators did not have comorbidity data. Whether a patient received EPP or P/D was very much based on how fit the patient was, so this introduced selection bias in the study. The authors were not able to support the use of one or the other surgical approach. They emphasized the need for additional well-designed prospective trials.

Okada et al. [23] are the only group from Japan reporting their experience with mesothelioma management. Their choice of whether to perform an EPP or a P/D was based on the degree of the tumour invasion into the lung as well as fitness of the patient for each surgical procedure. They used lung function tests to assess fitness. They concluded that older age, nonepithelial histology, and pathologic stages III-IV disease had a significantly negative impact on survival.

Their findings show that younger patients with a stages I-II epithelial tumour were good candidates for radical surgery, either P/D or EPP.

Aziz et al. [15] reported a retrospective review of their 10-year experience in their centre. P/D was considered for locally extensive disease but only for relief of pain or shortness of breath. The decision to perform an EPP as opposed to a P/D was based on how much the tumour had spread within the lung and whether patients were well enough to go through major surgery. During the more recent years they also decided not to operate on patients older than 60. This was because they believed that younger patients would be able to tolerate the operation better and therefore have better operative mortality results.

They did not find any significant difference in survival between patients undergoing P/D or having no surgery at all.

Several limitations exist related to the retrospective nature of the study. Systemic chemotherapy was not offered to patients who underwent P/D because this was essentially an attempt to reduce the tumour load in the lung, not to completely remove it, so chemotherapy seemed futile as an option.

Lang-Lazdunski et al. [24] in a ground-breaking and certainly controversial paper compared P/D with hyperthermic pleural lavage against EPP. This is the first prospective but not randomised study which is hinting on the possibility of treating patients with mesothelioma with P/D as a surgical curative attempt (complete resection). The authors did multivariate analysis and concluded that epithelioid histology, P/D, and completeness of resection were independent prognostic factors of completion of trimodality therapy. This means that patients who had P/D were well enough after the surgery to undergo chemotherapy and radiotherapy. Therefore they had a good chance of long-term survival. A significant limitation is the small sample size of this paper.

Rena and Casadio [25] published their single centre 11-year experience with EPP and P/D. Importantly they included a quality-of-life (QoL) assessment in their comparison. It is the only study of its kind and their findings agree with Lang-Lazdunski et al. [24] in supporting superiority of P/D over EPP.

QoL parameters were similar at baseline for patients undergoing either procedure but P/D patients had a better QoL at 6 and 12 months. All parameters were improved among P/D patients with the exception of postoperative pain, which was similar in the two groups. The authors thought that this was because both EPP and P/D use a posterolateral thoracotomy incision to get to the lung, which is associated with a lot of pain after the surgery.

Patients with lower stage MPM (I and II) who had P/D had a significantly better quality of life when compared with that of those submitted to EPP.

Patients with histological diagnosis of epithelial mesothelioma had similar survival regardless of which surgical procedure they had.

They also found that patients who underwent EPP had worse long-term survival than those who underwent P/D. This finding needs further exploration as in theory EPP achieves complete removal of MPM whereas P/D does not. Therefore we would expect EPP patients to live longer but the opposite was found in this study.

### 3.2. Pleurectomy/Decortication Only Studies

Nakas et al. [26] maintained a prospectively updated database which they used to analyse patients that underwent P/D only. These were patients unfit for EPP. Exclusion criteria that made them unfit for EPP were clinical stage T4 or M1 (cancer had extensive spread within the lung or had given metastases elsewhere in the body), mediastinoscopy proven stage N2 (cancer had spread to lymph nodes of the opposite side), age >70 years, poor lung function due to smoking or other lung problems (predicted postoperative FEV1 <40%), and right or left ventricular systolic dysfunction (poor myocardial contractility).

P/D was classified into radical (R) and nonradical (NR) depending on how extensive the resection of the pleura and tumour were. They found that patients undergoing radical P/D had a survival advantage over those who underwent nonradical P/D. There was a difference though in the distribution of the histological subtypes of the disease: there were proportionately more patients with epithelioid cell type (a less aggressive type) in group R (40 epithelioid, 4 sarcomatoid, and 7 biphasic) than in group NR (28 epithelioid, 11 sarcomatoid, and 12 biphasic).

In contrast to the majority of the other studies included in this review, Nakas et al. believe that with radical P/D they can achieve complete macroscopic clearance of tumour. The majority of investigators in other studies believe that only EPP can achieve clearance. Most surgeons think that P/D results in high rates of local recurrence of MPM.

An important limitation in their paper is that they did not have an established protocol regarding adjuvant treatment following P/D since these decisions were at the discretion of referring oncologists.

Martin-Ucar et al. [27] performed P/D for symptomatic control of patients diagnosed with MPM. Indications included shortness of breath, chest pain, and empyema (collection of pus in the chest cavity). Their treatment of patients with early stage disease was EPP. They were not part of this study. Radiotherapy and chemotherapy were not given to patients undergoing P/D unless there was clinical evidence of disease progression.

They found that at 3 weeks and 6 months after P/D the patients felt less breathlessness and less chest pain. However, despite good symptom control in those who survived, mortality outweighed the benefits after 3 months.

In multivariate analysis, an aggressive cell type and weight loss were predictors of poor symptom control. Patients with epithelial cell type and no weight loss were significantly more likely to retain symptomatic control than those with more aggressive cell type and loss of weight.

A recent paper by Lang-Lazdunski et al. [28] looked at patients unsuitable to undergo EPP or those that refused EPP. They developed an alternative multimodality therapy plan for these patients based on P/D and hyperthermic pleural lavage (hot water at 40°C mixed with Betadine was used to wash the pleural cavity) followed by prophylactic radiotherapy and adjuvant chemotherapy.

A large proportion of patients were referred to their hospitals for enrolment into the mesothelioma and radical surgery (MARS) trial [16] involving EPP during the 2005–2008 period. Those patients not wishing to undergo EPP or to enrol on the MARS trial were offered the alternative treatment described above. Selection bias is therefore an issue in this study as well as small sample size and nonrandomisation.

Completeness of P/D resection had a significant impact on survival. Those patients that had complete macroscopical removal of the MPM tumour had better survival than those who underwent an incomplete resection.

The authors concluded that treatment with P/D and hyperthermic pleural lavage was associated with low morbidity and mortality and therefore it could represent an alternative to the classical trimodality regimen.

### 3.3. Extrapleural Pneumonectomy Only Studies

Sugarbaker et al. [29] have published one of the largest series of patients treated with trimodality therapy for mesothelioma. This is one of the earliest publications on this topic and one of the most quoted papers that we encountered in our literature search.

Their conclusions at that time influenced the direction of research into trimodality regimes.

They found that multimodality therapy including EPP is feasible in selected patients, microscopic resection margins affect long-term survival and patients with epithelial subtype (less aggressive), margin-negative (complete removal of MPM with surgery), extrapleural node-negative (tumour not spread to lymph nodes elsewhere in the body) resection had extended survival. They also proposed a revision to the staging system that was used at that time and it has since been revised.

Krug et al. [30] conducted a multicentre phase II study in the US to assess performance of a treatment regime which consisted of neo-adjuvant chemotherapy, followed by EPP, followed by radiotherapy in patients with stages I to III disease. A rigorous assessment of each patient's fitness was done before enrolment to determine respiratory and cardiac function.

Among those who underwent EPP, median survival was 21.9 months. Among patients who completed radiotherapy, median survival was 29.1 months, 1-year survival was 90.0%, and 2-year survival was 61.2%.

Remarkably the parameters histological type of the tumour, gender, clinical stage of MPM, and lymph node spread did not influence survival.

Median time to relapse of mesothelioma was 18.3 months. Relapse-free rates among EPP patients were 63.8% at 1 year and 38.9% at 2 years.

One limitation of this study is that a mediastinoscopy to assess whether tumour had spread to lymph nodes outside the lung was not required for staging and data regarding how many patients underwent mediastinoscopy was not recorded. Furthermore these patients were highly selected on the basis of stage of disease (early stages), good performance status, and satisfactory heart and lung function. They were managed at centres that treat high volumes of patients with MPM.

The authors concluded that the treatment algorithm with induction chemotherapy, EPP, and then hemithoracic radiation is feasible and effective, but only a subgroup of patients experience long survival.

Buduhan et al. [31] did a retrospective review of 46 patients treated with trimodality therapy over a ten-year period in a single centre. Median survival for stages 0, II, III, and IV patients was 17, 33, 21, and 24 months, respectively. If the MPM involved lymph nodes, that was a poor prognostic finding, with a median survival of 11 months, compared with 34 months if node-negative. The authors estimate that, for every patient deemed acceptable of trimodality therapy, about another 4 mesothelioma patients were turned down because of advanced disease or comorbidities.

A limitation is that the choice of chemotherapy agent, dose, and schedule were at the discretion of the oncologist. During the last 4 years, the chemotherapy regimen was standardized to cisplatin and pemetrexed. Also patients were referred to EPP after they had already started on or completed chemotherapy at other institutions.

De Perrot et al. [32] conducted a retrospective analysis of 60 patients that were selected to undergo trimodality therapy for mesothelioma in Canada.

The chemotherapeutic regime was variable.

Disease-free survival was influenced by whether MPM had invaded lymph nodes or not (pathologic nodal status) and to a lesser extent by the histologic type of MPM. The median disease-free survival was 12 months for patients with N2 disease (MPM spread to lymph nodes outside the affected lung), 44 months for patients with N1 disease (MPM spread to lymph nodes of the affected lung), and not reached for patients with N0 disease (no lymph node involvement).

After multivariate analysis, the presence of N2 disease remained a significant prognostic marker of worse outcome despite completion of the trimodality therapy. Histological type, extent of lung invasion (T status), gender, and age were not significant.

Weder et al. [33] did a multicentre trial of patients undergoing trimodality therapy for mesothelioma and they assessed quality-of-life parameters as well as survival. Psychological distress was observed in all participants. The levels of distress did not change over time (from 0 to 6 months). There was a full recovery after a period of 6 months.

Roughly 20%–25% of patients experienced psychological problems. This reinforces the importance of supportive care interventions. Physical symptoms and activity showed worsening after surgery, but there was a recovery back to normal after 3–6 months. The authors concluded that QoL impairment was not a major issue; however, they stressed the importance of a specialised experienced team.

Rea et al. [34] published the latest phase II trial involving trimodality therapy. Only 22 patients managed to complete the full intervention including chemotherapy, EPP, and radiotherapy.

Following two cardiopulmonary-related deaths, the study protocol was changed. They reduced the total radiation dose administered to patients and included additional cardiopulmonary and respiratory function tests which led to a more careful selection of patients.

However this amendment made meaningful interpretation of results impossible as the baseline characteristics of the groups before and after the change in protocol were very different.

Van Schil et al. [35] published a study, which was original in that their primary endpoint was “success of treatment.” This was defined as a patient who received the full protocol treatment within the defined time frames and was still alive 90 days after the end of protocol treatment without progression or evidence of high toxicity related to the chemotherapy or the radiotherapy. Only 24 (42.1%) patients met the primary endpoint definition of success.

Median progression-free survival for all the 57 patients who were eligible and started treatment was 13.9 months and 1-year survival rate was 54.4%.

Trimodality treatment was completed in 37 (64.9%) patients and median treatment duration was 184 days.

Treasure et al. [16] embarked in a very ambitious project in 2005 to conduct a randomised controlled trial (MARS) across 12 centres in the UK, which would definitively determine the role of EPP in the management of mesothelioma in the context of trimodality treatment. They also included a quality of life assessment. They performed a state-of-the-art design trial.

12-month recurrence-free survival in the EPP group was 34.8% and median recurrence-free survival was 7.6 months.

Median quality-of-life scores seemed to be lower for the EPP group than the no EPP group, with the lowest median score shortly after surgery; however, there were no statistically significant differences between treatment groups.

At the time MARS was being planned, pemetrexed was not yet the standard of care in the UK. Unfortunately during recruitment, the chemotherapy standard of care for mesothelioma changed, and patients recruited later were more likely to receive cisplatin and pemetrexed than those recruited earlier in the study.

At the start of the trial the authors calculated that 670 patients would be needed to identify any statistically significant difference between EPP and no EPP with overall survival as the primary outcome.

Because of the anticipated difficulty in recruitment of such a high number of patients to a trial comparing EPP with a nonsurgical approach, the MARS researchers designed a feasibility trial with the objective of randomly assigning 50 patients within 1 year to assess the possibility of completing a larger trial to clarify the role of EPP. The study was therefore not designed to test the benefit of EPP for patient outcome and any conclusions were speculative. Moreover it could be argued that this feasibility study was partly unsuccessful, because it took 3 years to compile 50 patients.

Tonoli et al. [36] reviewed retrospectively 56 patients across three centres in Italy who had undergone EPP followed by different modalities of radiotherapy. Their primary purpose was to evaluate different radiotherapy strategies.

Eighteen patients (29.5%) had a recurrence during followup. The median time to recurrence was 10.7 months. Recurrent tumour (considered as the first site of relapse) was local in two cases (within the lung cavity), nodal in three cases (into regional or distant lymph nodes), and distant (metastasis in other organs) in 13 cases. The mean time from relapse to death was 5.2 months. Two patients survived for more than five years without evidence of MPM recurrence.

Tables 1 and 3 summarise study characteristics for P/D and EPP respectively.

Baseline characteristics, patient selection, and follow-up periods varied between institutions.

Tables 2 and 4 summarise survival and peri-operative outcomes for P/D and EPP, respectively.

For P/D median survival ranged from 8.1 to 32 months. Mortality ranged from 0% to 9.8% and morbidity ranged from 5.9% to 55%.

For EPP median survival ranged from 6.9 to 46.9 months. Mortality ranged from 3.2% to 12.5% and morbidity ranged from 10% to 82.6%.

This large variability makes it difficult to reach safe conclusions in relation to the efficacy and safety of each procedure and echoes the issues that I have discussed at the beginning of Section 3.

## 4. Discussion: Current and Future Research

### 4.1. EPP versus P/D

The principles of surgical management for cancer are similar for all types of solid tumours. Surgery for MPM aims at removing all the tumour that can be seen during the operation (macroscopic disease) whereas chemotherapy and radiotherapy aim to kill any remaining cancer cells locally (in the lung cavity) or in other organs (microscopic disease).

Two surgical techniques exist.

1. EPP involves resection of the ipsilateral (same side as the tumour) lung, visceral and parietal pleura (mesothelial linings of lung and chest wall), ipsilateral hemidiaphragm, and pericardium with reconstruction of the latter two structures to prevent cardiac and visceral herniation (movement of the heart or abdominal organs into the chest cavity after the lung has been removed). The description of the EPP surgical technique is common knowledge in the thoracic surgical community without much variation. Because of this uniformity in the definition of EPP, results from different hospitals can be compared, differences in outcomes can be studied, and meaningful conclusions can be reached.
2. P/D is an operation that has not been standardised yet. The reasons behind this are probably multifactorial. From the current review it has become clear that P/D was considered a sort of compromise procedure reserved for patients who were not fit enough from cardiovascular or respiratory point of view to undergo the definitive EPP procedure. However in recent years some surgeons tend to favour it over EPP mainly due to the fact that most studies have shown no superiority of EPP in terms of survival and also much more complications related to EPP.

The International Association for the Study of Lung Cancer Staging Committee (IASLC) recommended that the following terminology should be used in the Mesothelioma Staging Project.

- Extrapleural pneumonectomy (EPP): en bloc resection of the parietal and visceral pleura with the ipsilateral lung, pericardium, and diaphragm.
- Extended pleurectomy/decortication (EPD): parietal and visceral pleurectomy to remove all gross tumour with resection of the diaphragm and/or pericardium.
- Pleurectomy/decortication (P/D): parietal and visceral pleurectomy to remove all gross tumour without diaphragm or pericardial resection.
- Partial pleurectomy: partial removal of parietal and/or visceral pleura for diagnostic or palliative purposes but leaving gross tumour behind.

This will improve the researchers' ability to make meaningful comparisons between studies. In addition, this will also provide uniform descriptors to be used in future research and improve pathologic staging and efforts to provide accurate prognosis to patients [37].

Current evidence and the IASLC report led several International Mesothelioma Interest Group members (latest meeting in Boston 2012) to conclude that both surgical options (P/D or EPP) are valid as long as they aim at complete removal of the cancer. Furthermore they should be performed as part of a multimodality treatment for MPM.

They also agreed that EPP and P/D have different advantages and disadvantages. The choice of which one to use for each patient depends on the spread of MPM within the lung cavity, the preference and experience of the surgeon, and also which one is favoured at different hospitals.

Furthermore, it was collectively agreed that multimodality treatment should be performed in centers of high expertise and by surgeons who have achieved morbidity and mortality rates similar to what is reported in the current literature [38].

For the time being, there is no evidence-based answer as to which procedure—P/D or EPP—is the more appropriate technique to achieve long-term survival in patients with MPM. EPP has been reported to have better survival but comes at a higher cost of peri-operative mortality and morbidity. P/D is associated with not only less peri-operative risk, but also less long-term success in controlling the spread or recurrence of MPM.

The largest report comparing both procedures in a retrospective multicentre study on 663 patients, combining the experience of three large centers in the United States, concluded that the study emphasises the similarities in outcome after EPP or P/D [22].

The studies that were reviewed in this paper are highlighting the fact that nowadays the challenge is how to carefully select the patients that would benefit from each procedure (EPP or P/D). The timing of the operation and whether chemotherapy should come before or after surgery also vary between centres. The controversy around this issue remains and the debate in the scientific community is ongoing. There is one statement however that scientists seem to agree on: patients with histologically proven MPM and resectable tumour who could tolerate the three treatment modalities should be considered for a multimodal approach and be included in a trial if possible [38].

### 4.2. Chemotherapy

Despite more than two decades of intensive research into possible treatments for MPM, results have been disappointing. Only chemotherapy with cytotoxic agents has been proven to improve outcomes. A landmark randomised controlled trial with the acronym “Emphacis” demonstrated the superiority of cisplatin combined with pemetrexed in the management of patients with MPM. Survival increased from 9.3 to 12.1 months and this was also associated with quality-of-life benefit [39].

Since publication of this study the combination of cisplatin and pemetrexed has replaced all previous chemotherapeutic agents as the main therapy for patients who are having chemotherapy as part of multimodality therapy and for those who have too high risk to undergo a surgical procedure [40].

Previously, worldwide research into drug development used to rely on empirical testing of new agents in clinical trials. In vitro or in vivo molecular targets were used extensively.

In the current century, research has created a different route to discovery of new therapeutic drugs. It is now utilising our success in decoding the human genome. Genomic medicine was based on our efforts to identify targets which may have some clinical significance. The evolution of MPM involves a multistep carcinogenesis pathway. Some of the cell changes are called “passenger mutations” because although they are present they do not give a growth advantage to the cancer cell. These mutations can be targeted by new chemotherapeutic agents.

Unfortunately, no mutations of clinical value (that can be targeted by drugs or other interventions) have yet been identified in mesothelioma, and certainly none have been transferred into practical clinical application [40].

Experts have called for an international cooperative effort. Collection of tissue specimens is of paramount importance. These have the potential to give us more insight into identification of usable “passenger mutations” that can be targeted with new more efficient drugs.

### 4.3. Radiotherapy (RT)

Traditionally, adjuvant RT has been given through fields from in front of as well as behind the patient in order to include the hemithorax where the MPM is located in its entirety. The heart, liver, kidneys, and stomach are protected by using radiotherapy shields over them. However in this way the dose that the chest receives at the edges of the shields is uncertain. Therefore sometimes the radiologists end up giving too much or too little radiation to the chest.

New radiotherapy techniques have been developed in the past decade. Intensity-modulated radiation therapy (IMRT) is a complex technique that was designed to overcome the problems associated with the traditional technique. With IMRT normal organs are protected against receiving any radiation, and higher doses are delivered to the chest. Therefore this is safer for the patient and more precise way of delivering RT to the MPM. Moreover areas of underdosing or overdosing can be identified and corrected.

Current research focuses on arc therapy and helical tomotherapy which are rotational radiotherapy techniques that deliver radiation from more angles than IMRT. They visualise the target in three dimensions and this makes them ideal for mesothelioma. They have been shown to achieve good results in preventing spread of the MPM; however this comes with a cost: pneumonitis (reactive inflammation of the lung which has been radiated) is a common complication.

Research is ongoing in current trials to determine if theoretical advantages of new radiotherapy techniques can be translated into clinical benefit for mesothelioma patients [41].

## 5. Conclusion

This systematic review has shown that EPP and P/D for patients with MPM can be performed with an acceptable perioperative mortality rate in specialised centres. However, the evidence for long-term survival in patients operated-on in the context of TMT in the current literature is inconsistent. A number of prospective studies with standardised therapeutic strategies have reported relatively favourable outcomes. These encouraging results demonstrate the potential benefit that surgery can offer for patients treated by a multidisciplinary approach in specialist high volume centres.

## Figures and Tables

**Table 1:** Study characteristics: P/D.

Author/year	Center	Period	Design	Chemo	P/D	Rx	F/U
Schipper et al., 2008 [20]	Mayo clinic, USA	1985–2003	Retrospective review of prospective database	Unknown	44	Unknown	5 years
Luckraz et al. 2010 [21]	University Hospital of Wales, UK	1980–2010	Retrospective review of prospective database	All referred	90 of which 24 had TMT	All referred	5 years
Flores et al., 2008 [22]	3 cancer centres in USA	1990–2006	Retrospective review	Clinical trial	278	Clinical trial	5 years
Okada et al., 2008 [23]	Single centre, Japan	1986–2006	Retrospective review	None	34	None	
Nakas et al., 2008 [26]	Leicester, UK	10 years?when	Retrospective review	33 R and 13 NR	51 radical, 51 nonradical	33 R and 13 NR	4 years
Aziz et al., 2002 [15]	Scotland, single centre	1989–1998	Retrospective review	0	47	0	5 years
Martin-Ucar et al., 2001 [27]	Glenfield, UK	1997–2001	Prospective	0	51	0	1 year
Lang-Lazdunski et al., 2011 [28]	Guy's and St Thomas, UK	2004–2010	Prospective, observational	36	36	36	5 years
Lang-Lazdunski et al., 2012 [24]	Guy's and St Thomas, UK	2004–2011	Prospective, observational	54	54	54	5 years
Rena and Casadio 2012 [25]	Single centre, Italy	1998–2009	Retrospective	37	37	37	

**Table 2:** Survival and perioperative outcomes: P/D.

Author/year	Survival median	Disease-free survival	Mortality	Morbidity	Hospital stay	Comments
Schipper et al., 2008 [20]	Total P/D 17.2 months Subtotal P/D 8.1 months	TP, 1 year, 80%; 2 years 35% ST, 1 year, 30%; 2 years, 15%; 3 years, 3.7%	2.9%	2 + 2 subtotal (20 + 5.9%)	6.5 days (median)	Total or subtotal pleurectomy groups
Luckraz et al., 2010 [21]	26 months	55%, 2 years	1.1%	13%	unknown	
Flores et al., 2008 [22]	23 months	12% at 5 years	4%	6.4% (respiratory only reported)	unknown	
Okada et al., 2008 [23]	17	24%, 3 years	0%	unknown	unknown	
Nakas et al., 2008 [26]	15.3 R 7.1 NR	66%, 54%, 33%, and 16.3% at 1, 2, 3, and 4 years; R—45%, 16%, 4%, and 0%, NR	5.9 R—9.8% NR	55% R—28% NR	unknown	Unsuitable for EPP
Aziz et al., 2002 [15]	14	45% at 1 year; 0% at 3 years	0%	unknown	unknown	
Martin-Ucar et al., 2001 [27]	7.2	31% at 1 year	7.8%	9.8%	7	Target was symptom relief
Lang-Lazdunski et al., 2011 [28]	24	91.7% at 1 year; 61% at 2 years	0%	25%	unknown	TMT plus hyperthermic pleural lavage
Lang-Lazdunski et al., 2012 [24]	12.8	49% at 2 years; 30.1% at 5 years	0%	27.7%	unknown	
Rena and Casadio 2012 [25]	23 stage II 32 stage I	stage I: two years and 3 years, 67% and 44% stage II: 49% and 9%	0%	24%	7	QoL

**Table 3:** Study characteristics. EPP.

Author/year	Centre	Period	Design	Chemotherapy	EPP	Rx	F/U
Schipper et al., 2008 [20]	Mayo clinic	1985–2003	Retrospective review of prospective database	Unknown	77	Unknown	5 years
Luckraz et al. 2010 [21]	University Hospital of Wales, UK	1980–2010	Retrospective review of prospective database	All referred	49 of which 15 TMT	All referred	5 years
Flores et al., 2008 [22]	3 cancer centres in USA	1990–2006	Retrospective review	Clinical trial dependent	385 total	Clinical trial dependent	5 years
Okada et al., 2008 [23]	Japan single centre	1986–2006	Retrospective review	5	31	5	5 years
Aziz et al., 2002 [15]	Scotland single centre	1989–1998	Retrospective review	51	64	51	5 years
Lang-Lazdunski et al., 2011 [28]	Guy's and St Thomas, UK	2004–2011	Prospective observational	22	22	17	5 years
Krug et al., 2009 [30]	Multicentre, US	2003–2006	Randomised trial	77 neoadjuvant	54	44	5 years
Buduhan et al., 2009 [31]	Washington, USA	1997–2008	Retrospective	46 neoadjuvant	46	44	6 years
De Perrot et al., 2009 [32]	Single centre, Canada	2001–2007	Retrospective	60 neoadjuvant	45	30	5 years
Weder et al., 2007 [33]	Multicentre, Europe	2000–2003	Prospective	58 neoadjuvant	45	36	5 years
Rea et al., 2013 [34]	4 centres, Italy	2005–2010	Retrospective	52 neoadjuvant	41	32	2 years
Sugarbaker et al., 1999 [29]	3 centres, USA	1980–1997	Retrospective	183 adjuvant	183	183	5 years
Van Schil et al., 2010 [35]	11 centres, Europe	2005–2007	Prospective trial	58 neoadjuvant	42	37	3 years
Treasure et al., 2011 [16]	12 centres UK	2005–2008	RCT	24	19	8	2 years
Rena and Casadio 2012 [25]	Single centre, Italy	1998–2009	Retrospective	40	40	40	3 years
Tonoli et al., 2011 [36]	3 centres, Italy	2005–2010	Retrospective	48	56	56	6 years

**Table 4:** Survival and perioperative outcomes: EPP.

Author/year	Survival median months	Disease free survival	Mortality (30-day)	Morbidity	Hospital stay median	Comments
Schipper et al., 2008 [20]	16	1 year, 61%; 2 years, 25%; 3 years, 14%; 5 years, 9%	8.2%	50.7%	9 days	
Luckraz et al. 2010 [21]	19.5 months	24.5% at 2 years	8.2%	53%	unknown	
Flores et al., 2008 [22]	19 months	12% at 5 years	7%	10% (respiratory only reported)	unknown	
Okada et al., 2008 [23]	13	33% at 3 years	3.2%	unknown	unknown	
Aziz et al., 2002 [15]	13 35 for +chemotherapy subgroup	84% at 1 year; 48% at 3 years	9%	21%	unknown	
Lang-Lazdunski et al., 2011 [28]	12.8	18.2% at 2 years; 9% at 5 years	4.5%	68%	unknown	
Krug et al., 2009 [30]	16.8	65.2% at 1 year; 37.2% at 2 years	3.6%	2–10%	Unknown	
Buduhan et al., 2009 [31]	24 ITT	80% at 1 year; 60% at 2 years; 18% at 5 years	4.3%	2–7%	unknown	
De Perrot et al., 2009 [32]	14 ITT	60% 1 year 25% 2 years 10% 5 years	6.7%	33.3%	unknown	
Weder et al., 2007 [33]	19.8 ITT, 23 months EPP group	78% at 1 year; 45% at 2 years; 10% at 5 years	2.2%	35%	unknown	QoL assessment
Rea et al., 2013 [34]	6.9	33.3% at 1 year; 24.1% at 2 years	4.4%	66.7%	unknown	Protocol amended
Sugarbaker et al., 1999 [29]	19	38% at 2 years; 15% at 5 years	3.8%	50%	unknown	
Van Schil et al., 2010 [35]	18.4	70.2% at 1 year; 28% at 20 months	6.5%	82.6%	unknown	
Treasure et al., 2011 [16]	14.4	50% at 1 year	12.5%	62.5%	unknown	QoL assessed
Rena and Casadio 2012 [25]	18 stage II 28 stage I	31–57% at 2 years; 5–39% at 3 years, stage I or II	5%	62%	9	
Tonoli et al., 2011 [36]	46.9	Unknown	Unknown	Unknown	Unknown	
